# Free Radicals and Extrinsic Skin Aging

**DOI:** 10.1155/2012/135206

**Published:** 2012-02-29

**Authors:** Borut Poljšak, Raja Dahmane

**Affiliations:** ^1^Laboratory for Oxidative Stress Research, Faculty of Health Sciences, University of Ljubljana, Zdravstvena Pot 5, 1000 Ljubljana, Slovenia; ^2^Biomedicine in Health Care Division, Faculty of Health Sciences, University of Ljubljana, Zdravstvena Pot 5, 1000 Ljubljana, Slovenia

## Abstract

Human skin is constantly directly exposed to the air, solar radiation, environmental pollutants, or other mechanical and chemical insults, which are capable of inducing the generation of free radicals as well as reactive oxygen species (ROS) of our own metabolism. Extrinsic skin damage develops due to several factors: ionizing radiation, severe physical and psychological stress, alcohol intake, poor nutrition, overeating, environmental pollution, and exposure to UV radiation (UVR). It is estimated that among all these environmental factors, UVR contributes up to 80%. UV-induced generation of ROS in the skin develops oxidative stress, when their formation exceeds the antioxidant defence ability of the target cell. The primary mechanism by which UVR initiates molecular responses in human skin is via photochemical generation of ROS mainly formation of superoxide anion (O_2_^−^^∙^), hydrogen peroxide (H_2_O_2_), hydroxyl radical (OH^∙^), and singlet oxygen (^1^O_2_). The only protection of our skin is in its endogenous protection (melanin and enzymatic antioxidants) and antioxidants we consume from the food (vitamin A, C, E, etc.). The most important strategy to reduce the risk of sun UVR damage is to avoid the sun exposure and the use of sunscreens. The next step is the use of exogenous antioxidants orally or by topical application and interventions in preventing oxidative stress and in enhanced DNA repair.

## 1. Introduction

Human skin is naked and is constantly directly exposed to the air, solar radiation, other environmental pollutants, or other mechanical and chemical insults, which are capable of inducing the generation of free radicals as well as reactive oxygen species (ROS) of our own metabolism. A free radical can be defined as a chemical species possessing an unpaired electron [1]. It can also be considered as a fragment of a molecule. Free radicals, important for living organisms, include hydroxyl (OH^∙^), superoxide (O_2_^−∙^), nitric oxide (NO^∙^), thyl (RS^∙^), and peroxyl (RO_2_^∙^). Peroxynitrite (ONOO^−^), hypochlorous acid (HOCl), hydrogen peroxide (H_2_O_2_), singlet oxygen (^1^O_2_), and ozone (O_3_) are not free radicals but can easily lead to free radical reactions in living organisms. The term reactive oxygen species (ROS) is often used to include not only free radicals but also the nonradicals (^1^O_2_, ONOO^−^, H_2_O_2_, and O_3_). Reactive oxygen species are formed and degraded by all aerobic organisms, leading to either physiological concentrations required for normal cell function or excessive quantities, state called oxidative stress. Oxidative stress is the term referring to the imbalance between generating of ROS and the activity of the antioxidant defence [2]. Severe oxidative stress can cause cell damage and death.

ROS are usually of little harm if intracellular mechanisms that reduce their damaging effects work properly. Most important mechanisms include antioxidative enzymatic and nonenzymatic defences as well as repair processes. But the problem arises with age, when endogenous antioxidative mechanisms and repair processes do not work anymore in an effective way.

## 2. Free Radicals and Oxidative Stress

Extrinsic skin damage develops due to several factors: ionizing radiation, severe physical and psychological stress, alcohol intake, poor nutrition, overeating, environmental pollution, and exposure to UV radiation (UVR). UV-induced generation of ROS in the skin develops oxidative stress, when their formation exceeds the antioxidant defence ability of the target cell [3]. Acute exposure to UVR depletes the catalase activity in the skin and increases protein oxidation [4]. It is estimated that among all the environmental factors, UVR contributes up to 80% and it is the most important environmental factor in the development of skin cancer and skin aging [5]. The primary mechanism by which UVR initiates molecular responses in human skin is via photochemical generation of ROS mainly formation of superoxide anion (O_2_^−∙^), hydrogen peroxide (H_2_O_2_), hydroxyl radical (OH^∙^), and singlet oxygen (^1^O_2_) [6]. UVR penetrates the skin, reaches the cells, and is absorbed by DNA, leading to the formation of photoproducts that inactivate the functions of DNA. According to Pattison and Davies (2006), UVR can mediate damage via two different mechanisms: (a) direct absorption of the incident light by the cellular components, resulting in excited state formation and subsequent chemical reaction, and (b) photosensitization mechanisms, where the light is absorbed by endogenous (or exogenous) sensitizers that are excited to their triplet states. The excited photosensitisers can induce cellular damage by two mechanisms: (a) electron transfer and hydrogen abstraction processes to yield free radicals (Type I) or (b) energy transfer with O_2_ to yield the reactive excited state, singlet oxygen (Type II) [7]. Oxidation of DNA can produce different types of DNA damage: strand breaks, sister chromatid exchange, DNA-protein crosslinks, sugar damage, abasic sites, and base modifications. Cell death, chromosome changes, mutation, and morphological transformations are observed after UV exposure of prokaryotic and eukaryotic cells. Numerous types of UV-induced DNA damage have now been recognized that include stand breaks (single and double), cyclobutane-type pyrimidine dimers, 6–4 Pyo photoproducts and the corresponding Dewar isomer, thymine glycols, 8-hydroxy guanine, and many more.

Besides oxidation of nuclear DNA, UVR can induce also oxidative damage to mitochondrial DNA (mtDNA). Aging is a multifactorial phenomenon characterized by increased susceptibility to cellular loss and functional decline, where mitochondrial DNA mutations and mitochondrial DNA damage response are thought to play important roles [8]. It has been suggested that sunlight passing through the skin can even cause DNA damage in white cells circulating through skin capillaries [9], but the greatest damage is within the skin cells, including the damage to dermal mitochondrial DNA [10]. Singlet oxygen produced by UVA light has been shown to cause strand breaks in the mitochondrial DNA, which has resulted in mtDNA deletions. Mitochondrial DNA is believed to be the most critical target of endogenous ROS production since it lies in the inner mitochondrial membrane, in close proximity to the electron transport chain, where the most free radicals are formed. In the past, it was believed that mitochondria lack DNA repair capacity, but this is not true. The investigations performed in the last two decades have confirmed that mitochondria do possess effective DNA repair mechanisms, and the understanding of how these mechanisms function has significantly increased in the last few years. The main DNA repair pathway that was described to actively take place in mammalian mitochondria was the base excision repair or BER pathway [8]. However, it is true that mitochondria do not remove UV-induced DNA damage which might be important in photodamage and skin cancer formation. There have been observed greater accumulation of mtDNA found in sun-exposed skin compared to protected skin [11, 12]. The most frequent mutation is a 4,977-base pair deletion also called the common deletion, which is increased in photoaged skin. Although DNA damage due to ROS is not a rare event since it is estimated that human cell sustains an average of 10^5^ oxidative hits per day due to cellular oxidative metabolism [13], DNA is functionally very stable, so that the incidence of cancer is much lower than one would expect, taking into account the high frequency of oxidative hits.

## 3. Skin Antioxidants

The skin is equipped with a network of protective antioxidants. They include enzymatic antioxidants such as glutathione peroxidase, superoxide dismutase, and catalase, and nonenzymatic low-molecular-weight antioxidants such as vitamin E isoforms, vitamin C, glutathione (GSH), uric acid, and ubiquinol [14]. Various other components present in skin are potent antioxidants including ascorbate, carotenoids, and sulphydrils. Water-soluble antioxidants in plasma include glucose, pyruvate, uric acid, ascorbic acid, bilirubin and glutathione. Lipid soluble antioxidants include alphatocopherol, ubiquinol-10, lycopene, *β*-carotene, lutein, zeaxanthin, and alpha-carotene. In general, the outer part of the skin, the epidermis, contains higher concentrations of antioxidants than the dermis [15]. In the lipophilic phase, *α*-tocopherol is the most prominent antioxidant, while vitamin C and GSH have the highest abundance in the cytosol. On molar basis, hydrophilic non-enzymatic antioxidants including L-ascorbic acid, GSH, and uric acid appear to be the predominant antioxidants in human skin [16]. The stratum corneum (SC) was found to contain both hydrophilic and lipophilic antioxidants. Vitamins C and E (both *αγ* and *α*-tocopherol) as well as GSH and uric acid were found to be present in the SC [17, 18]. Surprisingly, they were not distributed evenly, but in gradient fashion, with low concentrations on the outer layers and increasing concentrations toward the deeper layers of the SC.

## 4. Conclusion

Skin DNA molecules are constantly “bombarded” by ROS originating from endogenous processes as well as from environmental agents and from radiation sources. Damaged DNA is being constantly repaired by many cellular repair systems. If the frequency of damaging events exceeds the repair capacity, damaged DNA is not repaired in time and can pass to daughter cells and thus trigger tumour initiation and progression process. Nevertheless, avoidance of excessive cumulative and sporadic sun exposure is important in reducing the risk of skin cancer and skin aging. Additionally, antioxidants might act by enhancing the DNA enzyme repair systems through a posttranscriptional gene regulation of transcription factors [19, 20]. Cellular antioxidant defence mechanisms are therefore crucial for the prevention or removal of the damage caused by the oxidizing component of UVR. Evidence is accumulating that dietary changes and special nutrients may help to reduce oxidative stress, free radical formation and thereby slow down the skin damage process. The primary treatment of photoaging is photoprotection, but secondary treatment could be achieved with the use of antioxidants and some novel compounds such as polyphenols. Exogenous antioxidants like vitamin C, E, and many others cannot be synthesized by the human body and must be taken up by the diet. They have been shown to prevent exogenous free radical formation (e.g., UVR). They could also possess beneficial effects in endogenous ROS prevention. Antioxidants can regulate the transfer of electrons or quench free radicals escaping from electron transport chain. Since the effectiveness of endogenous antioxidant system is diminished during aging, the exogenous supplementation of antioxidants might be a protective strategy against age-associated skin oxidative damage. It can be concluded that oxidative stress is a problem of skin cells, and endogenous as well as exogenous antioxidants could play an important role in decreasing it. However, it is important to pretreat the skin with antioxidants before sun exposure. Animal and human studies have convincingly demonstrated pronounced photoprotective effects of “natural” and synthetic antioxidants when applied topically before UVR exposure. No significant protective effect of melatonin or the vitamins when applied alone or in combination was obtained when antioxidants were applied after UVR exposure. UVR-induced skin damage is a rapid event, and antioxidants possibly prevent such damage only when present in relevant concentration at the site of action beginning and during oxidative stress [21]. Treatment of the skin with antioxidants after the damage was caused by UVR might cause additional harmful effects on cell cycle control and apoptosis process. The most important strategy to reduce the risk of sun UV radiation damage is to avoid the sun exposure and the use of sunscreens. The use of topical antioxidants is gaining favour among dermatologists because of their broad biologic activity. Many are not only antioxidants but also possess anti-inflammatory and anticarcinogenic activities. In general, topical antioxidants exert their effects by downregulating free-radical-mediated pathways that damage skin [22]. The next step is the use of exogenous antioxidants orally or by topical application and interventions in preventing oxidative stress and in enhanced DNA repair. A wide variety of antioxidants or other phytochemicals have been reported to possess substantial skin photoprotective effects, such as licopene, coenzyme Q, glutathione, carnosine, selenium, zinc, bioflavonoids, green tea polyphenols, grape seed proanthocyanidins, resveratrol, silymarin, genistein, and others on UV-induced skin inflammation, oxidative stress, and DNA damage.
